# High Variability of Postsurgical Anatomy Supports the Need for Individualized Drug-Eluting Implants to Treat Chronic Rhinosinusitis

**DOI:** 10.3390/life10120353

**Published:** 2020-12-17

**Authors:** Ziwen Gao, Farnaz Matin, Constantin Weber, Samuel John, Thomas Lenarz, Verena Scheper

**Affiliations:** 1 Department of Otorhinolaryngology, Head and Neck Surgery, Lower Saxony Center for Biomedical Engineering, Implant Research and Development (NIFE), Hannover Medical School, 30625 Hannover, Germany; Gao.Ziwen@mh-hannover.de (Z.G.); Matin.Farnaz@mh-hannover.de (F.M.); weber.constantin@mh-hannover.de (C.W.); Lenarz.Thomas@mh-hannover.de (T.L.); 2 Cluster of Excellence ‘Hearing4all’ EXC 1077/1, 30625 Hannover, Germany; 3 HörSys GmbH, 30625 Hannover, Germany; john.samuel@hoersys.de

**Keywords:** endoscopic sinus surgery, sinus anatomy, drug delivery, nasal implant, individualized frontal neo-ostium implant

## Abstract

Chronic rhinosinusitis (CRS) is a common disease in the general population that is increasing in incidence and prevalence, severely affecting patients’ quality of life. Medical treatment for CRS includes self-management techniques, topical and oral medical treatments, and functional endoscopic sinus surgery (FESS). FESS is a standard procedure to restore sinus ventilation and drainage by physically enlarging the inflamed sinus passageways. Nasal drug-releasing stents are implanted to keep the surgically expanded aperture to the sinus frontalis open. The outcome of such an intervention is highly variable. We defined the anatomical structures which should be removed, along with ‘no-go areas’ which need to be preserved during FESS. Based on these definitions, we used cone beam computed tomography (CBCT) images to measure the dimensions of the frontal neo-ostium in 22 patients. We demonstrate anatomical variability in the volume and diameter of the frontal sinus recess after surgery. This variability could be the cause of therapy failure of drug-eluting implants after FESS in some patients. Implants individually made to fit a given patient’s postsurgical anatomy may improve the therapeutic outcome.

## 1. Introduction

Chronic rhinosinusitis (CRS) with polyposis is a common health problem and one that affects sufferers’ quality of life. In Europe, CRS has a high prevalence of 10.9% (based on an epidemiological study [1]). CRS is defined by the presence of nasal and paranasal sinus symptoms for at least three consecutive months accompanied by objective evidence of inflammation of the mucosa of the nose and paranasal sinuses, in the form of either a computed tomography (CT) scan or a nasal endoscopy [2]. Patients with CRS typically report symptoms of respiratory and olfactory dysfunction, nasal congestion, nasal discharge, facial pain/pressure, and qualitative and quantitative loss of smell which can have a significant effect on health-related quality of life. In fact, the pathophysiological mechanisms of CRS are not fully clarified [3].

Sino-nasal topical treatments and systemic steroids are the first choice for relief of CRS symptoms and have been shown to be effective as an initial appropriate pharmacotherapy [4]. A variety of agents are commonly employed over a prolonged period, including corticosteroids, antimicrobials, and immune-modulating medications [2].

**Figure 1:**
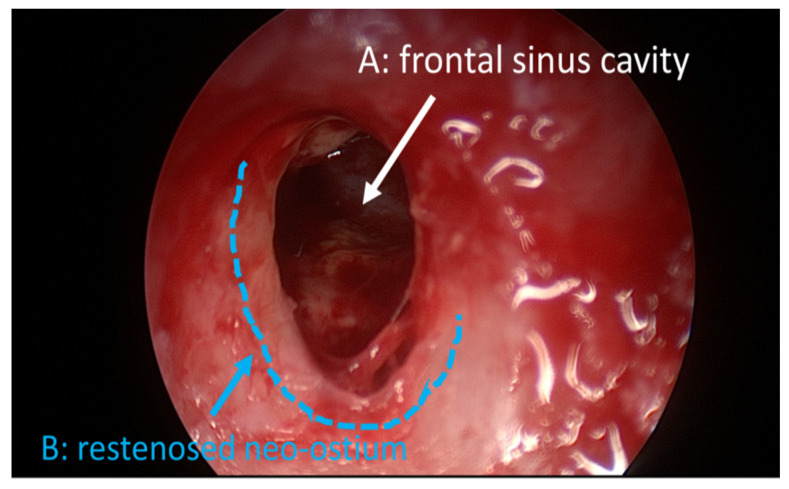
Intraoperative endoscopic appearance of the frontal sinus cavity following a nasalization procedure. (**A**) frontal sinus cavity; (**B**) re-stenosed neo-ostium.

For patients whose CRS is unresponsive to conservative treatment (no improvement after three consecutive months), the next option is functional endoscopic sinus surgery (FESS). FESS is used to open drainage pathways of the paranasal sinuses and facilitate medication delivery, and has now become the gold standard in surgical treatment for CRS [5]. The size of the frontal ostium is a key factor in frontal sinus drainage [6]. General guidelines for CRS therapy when considering FESS thus involve the removal of obstructing bone formation, mucous membranes, polyps and scar tissue within the sinus cavities, leading to the frontal neo-ostium which is created by the surgeon via removal of tissue and bone to open the airway into the frontal sinus. For all surgical procedures, a substantial amount of postoperative inflammation and swelling occurs, resulting in the formation of synechiae, and in restenosis (Figure 1); postoperative care is, therefore, a crucial factor in the success of FESS.

The postoperative regimens are various, including saline irrigation, nasal packing, topical or systemic steroids, topical decongestants, oral antibiotics, and/or sinus cavity debridement. A systematic review evaluating the evidence in favor of these therapies was published in 2017 by Eloy et al. The authors concluded that this evidence was not strong for any of these treatments, but also that there was a tendency to use nasal saline irrigation, topical nasal steroid spray, and sinus cavity debridement [5]. Furthermore, nasal packing is generally performed postoperatively. Simple dressings moistened with saline can be inserted manually following surgery. Foam dressings (e.g., SINU-FOAM™) are substances that form a gel when hydrated; they can be used for nasal packing to minimize bleeding and edema, and to prevent scar tissue or synechiae in the middle meatus for a short period of time. Middle meatal spacers are splint-like devices that keep the sinus cavities open post-FESS. There is some evidence that middle meatal spacers may reduce the formation of synechiae following FESS, although the available studies show significant heterogeneity in this outcome [7].

Not all FESS patients improve to a similar degree, and failures are still reported. Depending on the rigor with which success or failure is defined, failure rates of 15%–50% have been cited for sinus surgery [8]. One of the most common complications following FESS is the formation of scar tissue or synechiae [7], leading to revision surgeries. Frontal neo-ostium implants (FOIs) are intended to stabilize the sinus openings and the middle turbinates, reduce edema, and/or prevent obstruction by adhesions. After surgical intervention the infection and inflammation processes are ongoing, lasting for long durations of up to more than 12 weeks [9] and necessitating sustained local drug release over a lengthy period of time to increase the benefit derived from frontal sinus drainage. Drug-eluting paranasal sinus implants have increasingly been viewed as a new frontier in CRS management (for detailed review see [10]) because of their ability to preserve paranasal sinus patency by providing controlled, consistent drug release over a defined period of time to the sinus mucosa. Two commercially available drug-eluting spacers (Propel™ sinus implant (Intersect ENT); Relieva Stratus™ MicroFlow Spacer (Acclarent, Inc. CA, USA) can be used clinically for the treatment of CRS [11,12,13,14]. In clinical studies the devices demonstrate both efficacy and safety, but certain limitations still remain. For instance, the drug dose is fairly low (Propel™), and both the release period (Propel™ and Relieva Stratus™) and the period until the implant degrades (Propel™) are brief in comparison to wound healing processes. A drawback of the Relieva Stratus™ is that it has to be explanted, leading to resumption of the wound healing processes. Additionally, Propel™ is available in two sizes only (23 mm and 16 mm) and the Relieva Stratus™ comes in only one length, so that individual patient anatomy is not taken into consideration.

In summary, some medical devices have been employed to maintain patency of the paranasal sinuses postoperatively. Due to the significant heterogeneity in their clinical outcome, debate is still ongoing as to whether these devices actually improve health status following FESS. Amongst other factors such as drug load and insufficient duration of implant stability, individual patient anatomy may influence the biological effect that such devices have. Additionally, the complexity of the frontal ostium after FESS and the risk of injuring the orbital bone and skull base during insertion of an FOI have to be considered as factors affecting the safety and success of treatment of frontal sinus disease [6]. Analyzing radiological images from our clinical database, we investigated whether there is anatomical variability of the frontal neo-ostium between patients, which has to be taken into account when developing drug-eluting FOIs to treat CRS. For this purpose, we used preoperative DVT (digital volume tomography) scans and segmented the area which hypothetically could be the neo-ostium. Therefore, the segmentation of the area was performed for the purposes of planning the frontal neo-ostium, and not for measuring the real neo-ostium. To our knowledge this is the first study investigating the dimensions of the neo ostium of the sinus frontalis which may hypothetically be created by the surgeon during FESS and which may be kept open using a novel individualized drug eluting FOI.

## 2. Material and Methods

Dimensions of the frontal neo-ostium were obtained from cone beam computed tomography (CBCT) images of 22 patients (11 males and 11 females; 19–71 years of age) with the frontal sinus clearly visible in the Digital Imaging and Communications in Medicine (DICOM) data sets. Data were retrospectively collected from 225 patients visiting our clinic from 23 July to 25 November 2018 for rhinologic surgeries. The 225 relevant scans were conducted as part of routine clinical practice before any surgical intervention was performed. Patients with previous sinus or rhinologic surgery, malformations or ongoing CRS were excluded, resulting in 22 image data sets to be segmented and analysed for this study.

The study was conducted in accordance with the Declaration of Helsinki. Due to the retrospective design, no written information was given to the subjects in the study group. However, only patients who agreed to the general use of their data were selected. All patient data were anonymized prior to the retrospective analysis. The ethics committee of Hannover Medical School (MHH), Germany, approved the use of these patient data for the retrospective study (approval code: 1897-2013).

**Figure 2:**
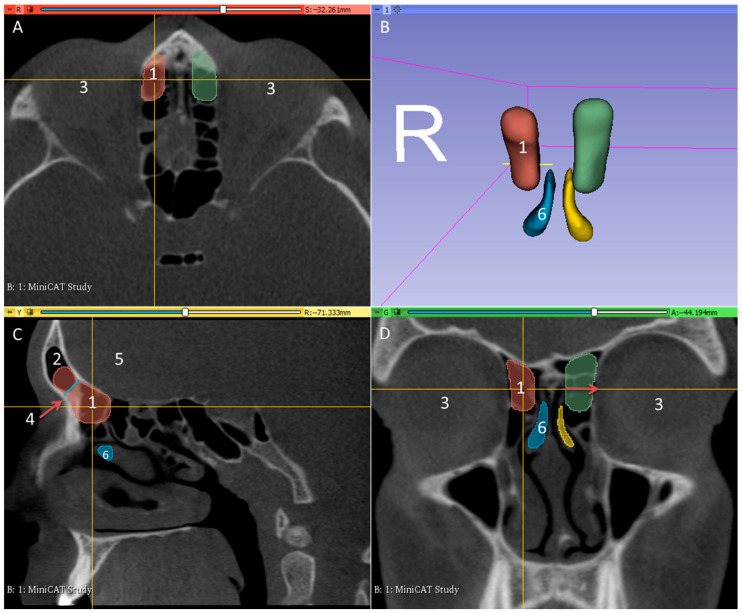
Example of the CBCT images used in this study. Axial (**A**), sagittal (**C**), and coronal (**D**) images at the level of the frontal sinus illustrate the frontal neo-ostium (arrows). The frontal sinus (**B**) is located superior to the medial eye socket and ethmoid sinus, with the sinus cavity between the medial and lateral bone plates behind the brow bow. Segmentation of the area for purposes of planning the frontal neo-ostium was performed at the frontonasal duct. 1: segmentation of area to be implanted on the right side; 3: eye; 4: frontal neo-ostium; 5: brain; 6: middle turbinate.

The patients were scanned using a 3D Accuitomo 170 digital CBCT scanner (J. Morita Tokyo mfg. Corp., Tokyo, Japan). Resulting CBCT volume images were reconstructed and exported in DICOM files using i-Dixel (J. Morita Tokyo Mfg. Corp., Tokyo, Japan) with a voxel size of 0.08 mm^3^. Segmentation of the DICOM data was performed using the 3D Slicer^TM^, version 4.11 (http://www.slicer.org) [15]. The area of the frontal neo-ostium was segmented manually, using a special tool for filling between slices, by a single experienced ENT physician at MHH. In order to standardize the head position for measurements, sagittal and axial planes were defined by anatomical landmarks as demonstrated in Figure 2. The frontal neo-ostium was defined on the parasagittal image by a line in blue drawn from the nasofrontal beak to the anterior skull base, represented by the junction of the anterior wall and inferior wall of the anterior cranial fossa (Figure 2C). The anterior wall begins at the posterior wall of the agger nasi region. The posterior wall is bound by the anterior wall of the ethmoid bulla, extending to the boundary of the anterior cranial fossa and alongside the skull base to the ethmoidal foramen. The lateral boundary is the middle turbinate, the outer boundary is the lamina papyracea, the medial border runs from the middle turbinate to the lamina papyracea, and the superior line is the ethmoid roof. The boundaries of the segmented frontal neo-ostium follow the landmarks report by Ting et al., 2014 [16].

Cross-sectional measurement of the neo-ostium was carried out on every scanned slide of the pre-operative scan. After completing the segmentation in the slice views, the implant surface was processed by applying the surface smoothing effect with the dimensionless parameter of 0.5 using the 3D Slicer^TM^, because this value yielded a good visual compromise between preservation of the segmented boundary and removal of sharp edges and spikes. All of the models were transformed into a hollow object and exported as a standard tessellation language (STL) file for further processing. The frontal neo-ostium models were measured to obtain surface area and volume using the tools available with the 3D Slicer^TM^. To determine the diameter of the frontal neo-ostium’s entrance, the distance was measured between the level of the middle turbinate and the half-way point of the lamina papyracea, as shown in Figure 2D (red arrow).

## 3. Statistical Analysis

The surface area, volume and diameter of the frontal neo-ostium were analysed using Prism 5.02 (GraphPad Software, Inc., La Jolla, CA, USA). Data were checked for normal distribution using the D’Agostino & Pearson normality test. The mean ± the standard deviation (SD) of the measured data were compared using the unpaired two-tailed *t*-test, as were grouped data for sex. The mean ± SD of the volume and surface area were correlated to age using the Spearman correlation test. Statistical significance was considered at *p* values less than 0.05.

## 4. Results

**Figure 3:**
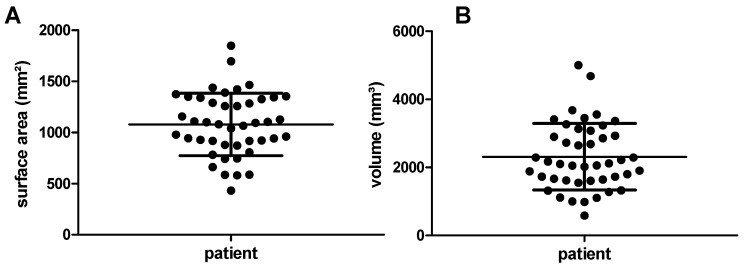
Graphical illustration of the frontal neo-ostium’s surface area and volume. (**A**) The average surface area of the 22 frontal neo-ostia analyzed is 1080 ± 306mm^2^. (**B**) The average volume is 2311 ± 947 mm^3^. For both measured parameters, high interpatient variability—more than 1400 mm^2^ (surface area) and more than 4000 mm^3^—was observed.

The surface area of the frontal neo-ostium in all patients—with two structures analyzed per patient (left and right side)—varies between 430 mm^2^ and 1849 mm^2^ (minimum to maximum), with a mean area of 1080 mm^2^ ± 306 mm^2^ (Figure 3A). The frontal neo-ostium’s volume ranges from 584 mm^3^ to 5001 mm^3^ (minimum to maximum), the mean volume being 2311 mm^3^ ± 974 mm^2^ (Figure 3B).

**Figure 4:**
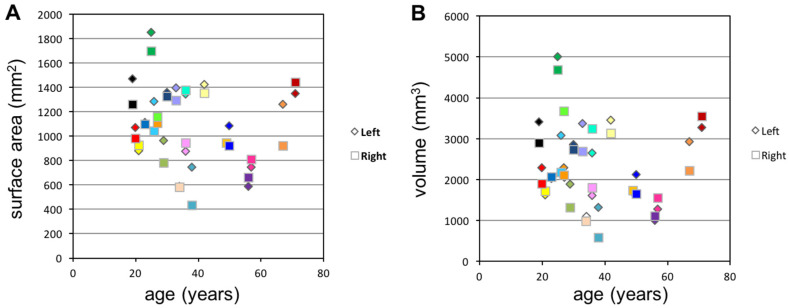
Surface area and volume of frontal neo-ostium in relation to age (bilateral data). Squares and diamonds represent the right and left sides, respectively. The same color is used for the two sides in a given patient. (**A**) The frontal neo-ostium surface area exhibits high variability which does not correlate with age. (**B**) The volume of the frontal neo-ostium show significant differences between individuals and are not corresponding with age. There are also some patients in whom the left and right segmented areas differ massively in volume and surface area.

The segmented areas and volumes of the left and right frontal neo-ostium within the same individual are plotted against patient age in Figure 4. There was no correlation between the frontal sinus neo-ostium’s surface area or volume and the age of the patient. The graph illustrates that, in some cases, there were massive differences in the neo-ostium within a given individual.

**Figure 5:**
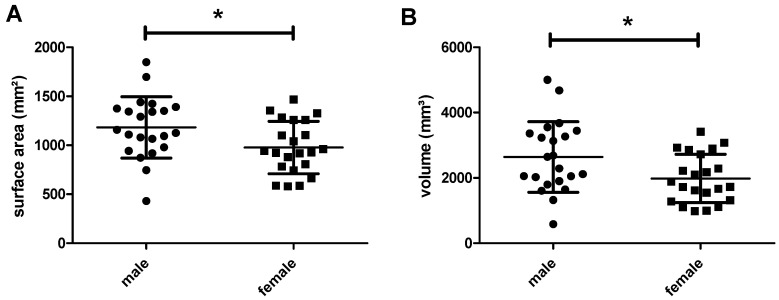
Segmented surface area (**A**) and volume (**B**) of the frontal neo-ostium: comparison between male and female patients. Both measures are significantly larger in male than in female patients. Results denoted with * (*p* < 0.05) were significant.

Grouping the measured data by sex, and analyzing this data using the t-test, reveals that the frontal neo-ostium’s surface area and volume are significantly larger in male than in female patients (Figure 5). Average surface area in male individuals was 1183 ± 66 mm^2^; in female patients, it was 976 ± 56 mm^2^ (*p* = 0.0237). With reference to the frontal neo-ostium’s volume, similar sex-specific differences were observed: 2640 ± 230 mm^3^ in male patients and 1983 ± 157 mm^3^ in female individuals (*p* = 0.0234).

**Figure 6:**
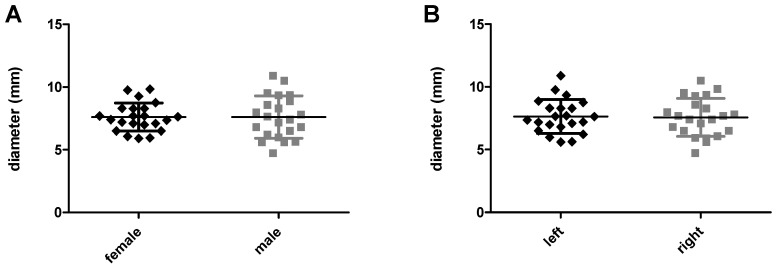
Comparison of the frontal neo-ostium diameter in female and male patients (**A**) and within patients (**B**). The mean diameter did not differ significantly between male and female individuals and between the left and right side.

For an FOI, the entrance of the frontal neo-ostium is important since its diameter determines the possible outer diameter of the implant. Comparing neo-ostium diameter between male and female patients yields no statistically significant differences, the mean ± SD being 7.606 mm ± 1.12 mm and 7.605 mm ± 1.68 mm (*p* = 0.9983), respectively (Figure 6A). In both sexes the median is 7.60 mm. In female patients, the minimum and maximum diameters are 5.91 mm and 9.83 mm; in male individuals, these diameters are 4.73 mm and 10.9 mm, respectively. The diameter of the left and right side ranges from 5.61 mm to 10.9 mm and 4.73 mm to 10.5 mm, respectively (minimum to maximum in each case), with a mean ± SD diameter of 7.641 mm ± 0.2874 mm (left side) and 7.570 mm ± 0.3226 mm (right side) (*p* = 0.8704) (Figure 6B).

**Figure 7:**
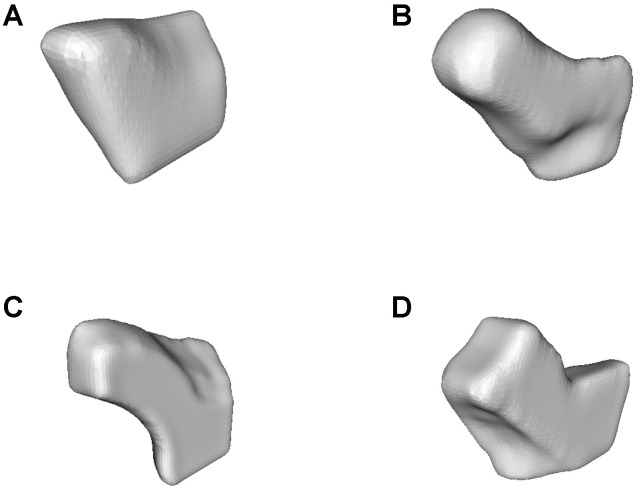
Examples of segmented 3D model. The volume is a function of the outer surface area and the shape of the frontal neo-ostium. Strong variability in shape is observed, both between patients and within a given patient. All segmentations were manually performed by a single trained ENT physician at Hanover Medical School (MHH). (**A**,**B**) The images show how the frontal neo-ostium differs in shape between the left and right side in the same patient. (**C**,**D**) These examples of the frontal neo-ostium in another patient exhibit a very great difference as compared with the first patient (compare images (**A**,**B**)).

Segmentation of the anatomical structures revealed strong variability in shape (Figure 7). The structure to be implanted is more or less straight in some cases and tends to be more angled in others.

## 5. Discussion

Although a large number of studies have shown that functional endoscopic sinus surgery (FESS) is a safe and effective treatment for chronic rhinosinusitis (CRS), its therapeutic mechanism is not well understood [17] and the outcome is of great variability. To improve the surgical technique, computer assisted navigation can be useful for the most experienced surgeons to decrease the recurrence rate and to reduce the total nasal resistance [18], and novel implants keeping the airway open may postoperatively improve the therapy.

The frontal ostium is a cavity present inside the frontal bone; of the paranasal sinuses, it is the one that is of most interest and significance in forensic identification due to its irregular shape and because of individual characteristics which make the frontal bone unique to every individual [19]. Using the segmentation technique described above we found strong variability in patients’ frontal neo-ostium, which is the hollow structure remaining after the physician has surgically cleared the frontal recess in patients suffering from CRS. Based on our data, males exhibit greater variability of the surface area and volume of the frontal neo-ostium, and their frontal neo-ostium is, on average, larger in terms of surface area and other size measures than that of females (Figure 6). This matches the findings of Camargo et al. [20] who reported that the mean total area of the frontal sinus, in general, without surgical intervention as considered in our analysis, was greater in males. However, in contrast to Camargo et al. who report the left area as being larger than the right area, we did not find a side-specific difference for neo-ostium volume, surface area or diameter. In addition, the above-mentioned study states that the frontal sinus parameters change with age. In our study, the surface area and volume of the frontal neo-ostium are not age dependent (Figure 5).

**Figure 8:**
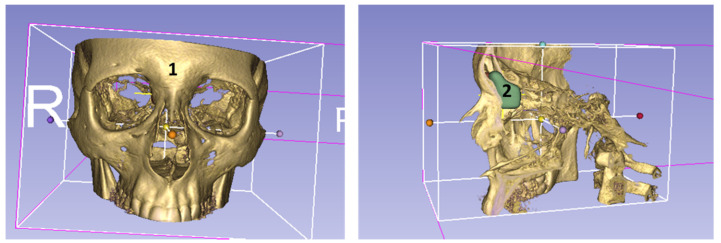
Reconstruction of a DVT-image data set illustrating in a coronal (**left image**) view the anatomical position of the sinus frontalis (1) and the area to be implanted with a FOI (2) in the sagittal (**right image**) view.

Based on the individual anatomy and the great variability of paranasal structures, and the risk of traumatizing sensitive structures like the brain and eye, we conclude that novel individualized drug-releasing implants should be developed. 3D slicing software packages digitally ‘slice’ a DICOM file into layers suitable for 3D printing, which may be one technique for generating drug-releasing individualized FOIs. The slicing process can readily be performed using proprietary software provided with commercially available 3D printers which usually possess a simple graphic user interface, such as Cube Software (3D Systems, Rock Hill, SC, USA) and MakerBot Desktop (MakerBot Industries, New York, NY, USA) [21]. In our study, the tool filling between slices allows an easy to obtain correct threshold adjustment in each scan data set; however, this may result in the segmented volume having inconsistent boundaries, leading to the segmented structure appearing more strongly variable than it is in reality. The segmentation technique described above may, therefore, indicate an approximate range for the FOI rather than the exact dimensions (Figure 8). To overcome this aspect (which is true for all segmentations), image acquisition protocols optimized for post-processing are under development. These tools and methods will improve segmentation quality in future, but require careful validation before being used in the context of clinical trials [22].

In order to be appropriate, manufacturing options for a personalized FOI have to address the individual patient’s requirements with respect to anatomy as well as pharmaceuticals. In this regard, additive manufacturing (AM)—also referred to as 3D printing—offers great potential. AM not only allows the rapid realization of geometrically complex medical implants suited to a patient’s individual anatomical needs, but also the individualized drug-loading of implants [23,24]. AM involves automatic layer-by-layer deposition of materials based on digital information. There is a multitude of AM processes, such as fused deposition modeling (FDM), Digital Light Processing (DLP) and stereolithography (SLA), that differ in terms of parameters including processable materials, solidification process and 3D printing resolution. Consequently, there are a large number of manufacturing options for FOI that utilize additive manufacturing technology.

However, availability of biocompatible materials suitable for implants is still very limited. Based on this, it becomes clear that different AM techniques offer different opportunities but also pose different challenges.

## 6. Conclusions

Chronic rhinosinusitis (CRS) is a highly prevalent and heterogeneous condition. Surgery considerably affects sufferers’ anatomy and has a host of knock-on effects on aspects such as wound healing, mucosal function, and the microbial milieu in the postoperative sinus cavities. Manual segmentation and measurement of the surface area and volume of the frontal neo-ostium in 22 patients confirms that there is strong variability both between and within individuals in the shape and volume of the frontal neo-ostium after surgical intervention. This variability influences the outcome and should be considered when developing novel drug-eluting frontal neo-ostium implants (FOIs). We hypothesize that these anatomical variabilities of the frontal neo-ostium, which are not taken into account in today’s FOIs, may be one factor affecting the treatment impact of CRS using drug-eluting frontal sinus implants. Individualized pharmaceutical frontal neo-ostium implants need to be developed and may be manufactured using additive technologies.
